# *Alpinia zerumbet*‐Derived Extracellular Vesicles Protect Against UVB‐Induced Photoaging by Enhancing Mitochondrial Bioenergetics and Skin Barrier Function

**DOI:** 10.1002/biof.70134

**Published:** 2026-07-03

**Authors:** Mo‐Rong Xu, Hui‐Chun Wang, Meng‐Shiou Lee, Sheng‐Yang Wang

**Affiliations:** ^1^ Department of Forestry National Chung‐Hsing University Taichung Taiwan; ^2^ Graduate Institute of Natural Products, Kaohsiung Medical University Kaohsiung Taiwan; ^3^ Department of Chinese Pharmaceutical Science and Chinese Medicine Resources China Medical University Taichung Taiwan; ^4^ Program in Specialty Crops and Metabolomics, Academy of Circular Economy National Chung Hsing University Nantou Taiwan; ^5^ Agricultural Biotechnology Research Center, Academia Sinica Taipei Taiwan

**Keywords:** *Alpinia zerumbet*, extracellular vesicles, HaCaT, mitochondrial biogenesis, oxidative stress, tight junction proteins, ultraviolet B (UVB) radiation

## Abstract

Ultraviolet B (UVB) radiation is a major cause of skin photoaging by oxidative stress, mitochondrial dysfunction, and barrier disruption. Plant‐derived extracellular vesicles (PDEVs) have emerged as promising bioactive nanocarriers, but their roles in mitochondrial regulation and skin barrier homeostasis remain unclear. This study aimed to investigate the protective effects of extracellular vesicles derived from *Alpinia zerumbet* leaves (AZEVs) against UVB‐induced photoaging in HaCaT keratinocytes. The results indicated that AZEVs exhibited typical extracellular vesicle characteristics, with an average size of 150 nm and a negative surface charge. Functionally, AZEVs significantly enhanced cell viability in UVB‐exposed HaCaT cells, reduced ROS accumulation, and restored mitochondrial membrane potential and ATP production. Moreover, AZEVs upregulated mitochondrial biogenesis‐related signaling pathway proteins (SIRT1, PGC‐1*α*, Nrf2, and *p*‐AMPK/AMPK) and TJ protein expression (ZO‐1, occludin, and claudin‐3), preserving barrier integrity and promoting wound healing. In addition, AZEVs are enriched in conserved miRNAs (miR166, miR159, and miR156) and a novel miRNA (novel_1), with predicted targets involved in redox regulation and energy metabolism. In summary, AZEVs protect keratinocytes against UVB‐induced damage by enhancing mitochondrial function, reducing oxidative stress, and preserving skin barrier integrity. These findings highlight AZEVs as a promising natural nanoplatform for anti‐photoaging and skin repair applications.

## Graphical abstract

AZEVs derived from *Alpinia zerumbet* leaves protect HaCaT keratinocytes from UVB‐induced photoaging by reducing reactive oxygen species (ROS) accumulation, restoring mitochondrial membrane potential, and enhancing ATP production. AZEVs upregulate mitochondrial biogenesis–related proteins, including *p*‐AMPK/AMPK, SIRT1, PGC‐1*α*, and Nrf2, thereby improving mitochondrial function and cellular antioxidant capacity. Furthermore, AZEVs preserve epidermal barrier integrity by maintaining tight junction proteins such as ZO‐1, occludin, and claudin‐3, which are otherwise disrupted by UVB exposure. Small RNA profiling indicates that AZEVs are enriched with conserved and novel miRNAs, including novel_1, which potentially regulate genes involved in redox balance and energy metabolism. Overall, AZEVs mitigate UVB‐induced skin cell damage through combined effects on oxidative stress suppression, mitochondrial bioenergetics enhancement, and barrier protein stabilization, highlighting their potential as natural nanocarriers for anti‐photoaging and skin repair. Figure created using BioRender.com.

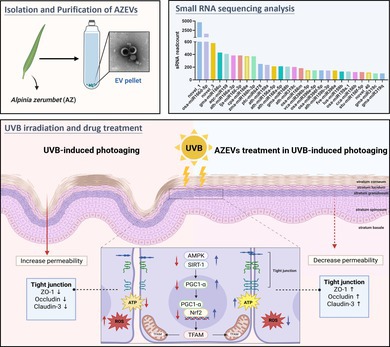

## 1 Introduction

The skin, as the largest organ of the human body, serves not only as a critical barrier against external environmental insults but also plays an essential role in maintaining water homeostasis and immune defense. Chronic exposure to ultraviolet (UV) radiation, particularly UVB, leads to skin photoaging [1], which is characterized by collagen degradation, disruption of epidermal barrier function, and increased oxidative stress [2, 3]. Emerging evidence indicates that mitochondrial dysfunction plays a pivotal role in UVB‐induced skin aging. UVB exposure can induce mitochondrial DNA damage, reduce ATP production, and promote the accumulation of reactive oxygen species (ROS), thereby impairing cellular energy metabolism and signaling pathways [4, 5]. Conversely, promoting mitochondrial biogenesis, particularly through the regulation of the SIRT‐1/PGC‐1*α*/TFAM signaling axis, has been recognized as an effective strategy for maintaining cellular energy homeostasis and mitigating photoaging [6, 7].

In addition to mitochondrial function, the integrity of the skin barrier is another critical factor in maintaining skin health. The skin barrier relies on a multilayered structure composed of the stratum corneum and intercellular tight junctions (TJs) [8]. TJs are primarily localized in the stratum granulosum and play an essential role in regulating transepidermal water loss (TEWL) and preventing the penetration of external substances. Key tight junction proteins, including ZO‐1, occludin, and claudin‐1, are crucial for maintaining epidermal barrier structure and function [9]. UVB exposure has been shown to disrupt the expression of these barrier‐associated proteins, leading to impaired skin barrier function and increased inflammatory responses [10, 11]. Recent evidence further suggests that preserving mitochondrial structure and function can mitigate UVB‐induced damage, enhance cell viability, and promote the restoration of skin barrier integrity [4, 12]. Therefore, the coordinated regulation of mitochondrial energy metabolism and skin barrier integrity has emerged as a promising strategy for preventing or delaying photoaging.

Extracellular vesicles (EVs) are nanoscale lipid bilayer vesicles secreted by various cell types and are widely present in biological fluids such as blood, urine, saliva, and cell culture media [13]. Based on their biogenesis and size, EVs are generally classified into exosomes, microvesicles, and apoptotic bodies. Among these, exosomes are enriched with proteins, lipids, and nucleic acids, enabling them to mediate intercellular communication and regulate diverse physiological processes [14]. In addition to mammalian cells, plants also release EV‐like nanoparticles, referred to as plant‐derived extracellular vesicles (PDEVs) or plant exosome‐like nanoparticles (PELNs). These vesicles typically range from 30 to 150 nm in diameter and share structural and compositional similarities with mammalian exosomes, including a lipid bilayer and diverse biomolecular cargos such as proteins, lipids, and nucleic acids [15]. Recent studies have demonstrated that PDEVs exhibit excellent biocompatibility and molecular transport capacity, allowing them to deliver bioactive molecules and modulate mammalian cellular functions, including antioxidant, anti‐inflammatory, and tissue repair effects [16, 17]. Owing to their natural origin and diverse bioactivities, PDEVs have attracted increasing attention in regenerative medicine and dermatological applications. Notably, accumulating evidence indicates that these vesicles can promote collagen synthesis, alleviate oxidative damage, and support skin regeneration, highlighting their potential as natural alternatives to synthetic ingredients for anti‐aging and skin barrier enhancement [18, 19]. However, the molecular mechanisms underlying their roles in regulating mitochondrial function and maintaining skin barrier integrity remain largely unclear.

*Alpinia zerumbet* , a member of the Zingiberaceae family, is widely distributed in East and Southeast Asia and has long been utilized in traditional medicine and daily diets. Its leaves possess significant ethnobotanical value across various regions. In Taiwan, the leaves are commonly used as natural wrappers for traditional rice dumplings [20]. In Okinawa, Japan, the leaves are used in traditional *mochi* (rice cakes wrapped in *A. zerumbet* leaves) and are believed to help prevent colds; they are also consumed as herbal teas [21]. In addition, essential oils extracted from the leaves have been widely applied in cosmetics, perfumes, insect repellents, and deodorants [22]. These traditional applications suggest that *A. zerumbet* leaves possess diverse bioactive potential. Phytochemical analyses have identified various functional compounds, including flavonoids, terpenoids, and polyphenols, which exhibit antioxidant, anti‐inflammatory, and anti‐aging properties [23]. Despite these promising bioactivities, studies on extracellular vesicles derived from *A. zerumbet* , particularly from its leaves, remain limited. Moreover, their potential roles in regulating UVB‐induced photoaging, mitochondrial energy metabolism, and skin barrier integrity have yet to be fully elucidated.

Therefore, this study aimed to isolate extracellular vesicles derived from *Alpinia zerumbet* leaves (AZEVs) and investigate their protective effects against UVB‐induced photoaging in HaCaT keratinocytes. We further evaluated whether AZEVs could alleviate UVB‐induced cellular damage by promoting mitochondrial biogenesis and maintaining the expression of skin barrier‐related proteins. This study is expected to provide mechanistic insights into the role of PDEVs in regulating cellular energy metabolism and skin barrier function, and to offer a scientific basis for the development of natural anti‐photoaging strategies.

## 2 Materials and Methods

### 2.1 Chemicals and Reagents

High‐glucose DMEM (Gibco, USA; 12100‐046), fetal bovine serum (Gibco, USA), 100 mM sodium pyruvate (Corning, USA; 25‐000‐CI), penicillin‐streptomycin (Gibco, USA; 15140‐122), ATP detection assay kit‐luminescence (Cayman Chemical, USA), DMSO (Sigma‐Aldrich, Germany; D2650), methanol‐d_4_ (Sigma‐Aldrich, Germany; 151947‐10G‐GL), deuterium oxide (Sigma‐Aldrich, Germany; 151882‐100G), 3‐(trimethylsilyl)propionic‐2,2,3,3‐d_4_ acid sodium salt (TSP) (Sigma‐Aldrich, Germany; 269913‐1G), Enhanced Cell Counting Kit‐8 (Elabscience, China; E‐CK‐A362), Pierce RIPA buffer (Thermo Fisher Scientific, USA), protease inhibitor cocktail (GoalBio, Taiwan; HC100‐007), phosphatase inhibitor cocktail (GoalBio, Taiwan; HC100‐008), bovine serum albumin (Gibco, USA), Bio‐Rad protein assay dye reagent concentrate (Bio‐Rad, USA), 5× protein sample dye (GeneMark, Taiwan; GM47‐b), mitochondrial membrane potential assay kit (JC‐1) (Abbkine, China; KTA4001), L‐amino acids (Sigma‐Aldrich, USA; LLA21), resveratrol (≥ 99% HPLC, Sigma‐Aldrich, Germany; R5010), H_2_DCFDA (2′,7′‐dichlorofluorescin diacetate) (Sigma‐Aldrich, Germany; D6883).

### 2.2 Isolation and Purification of AZEVs

The fresh *Alpinia zerumbet* leaves used in this study were collected (500 g) from the campus of National Chung Hsing University (GPS, 24°07′21.8″ N 120°40′39.4″ E) in August 2025 and were identified by Prof Yen Hsueh Tseng (Department of Forestry, National Chung Hsing University, Taichung, Taiwan). The voucher specimen (TCF Tseng4568) was deposited in the herbarium of the same university. Approximately 500 g of fresh plant material was thoroughly washed, cut into small pieces, and homogenized in ice‐cold phosphate‐buffered saline (PBS) at a 1:2 (w/v) ratio using a blender. The homogenate was first filtered through gauze and subsequently subjected to a second filtration using a Büchner funnel to remove large debris. The filtrate was aliquoted and stored at −80°C until further processing. For extracellular vesicle isolation, samples were thawed and subjected to sequential differential centrifugation at 4°C. Briefly, the homogenate was centrifuged at 3000 × *g* for 30 min using a high‐speed centrifuge (Model 6000, Kubota Corporation, Japan) to remove cell debris and nuclei, followed by centrifugation at 10,000 × *g* for 30 min to eliminate organelles and large vesicles. The resulting supernatant was sequentially filtered through 0.45 and 0.22 μm filters to remove particles larger than extracellular vesicles. The filtrate was then transferred to ultracentrifuge tubes and centrifuged at 150,000 × *g* (approximately 28,500 rpm) for 90 min at 4°C using an ultracentrifuge (Optima L‐90K, Beckman Coulter, USA). After centrifugation, the supernatant was carefully discarded, and the pellet was resuspended in 1 mL of double‐distilled water to obtain *A. zerumbet* leaf‐derived extracellular vesicles (AZEVs). The vesicle suspension was further filtered through a 0.22 μm syringe filter and stored at −80°C for subsequent experiments.

### 2.3 Nanoparticle Tracking Analysis (NTA) Analysis

The concentration and size distribution of plant‐derived extracellular vesicles were analyzed by the Instrumentation Center of Kaohsiung Medical University. Nanoparticle tracking analysis (NTA) was performed using a ZetaView particle analyzer (Particle Metrix GmbH, Germany) to determine particle size distribution and concentration.

Before analysis, the vesicle suspension was exchanged into PBS to ensure a stable ionic environment, and the suspension was subsequently diluted 1000‐fold in PBS. The samples were measured at 25.3°C, and videos were recorded at 11 predefined positions, with each position scanned three times. The camera sensitivity was set to 85, and the shutter speed was fixed at 100. The mean particle size, particle size distribution, and particle concentration were calculated from the recorded data.

### 2.4 Dynamic Light Scattering (DLS) and Zeta Potential Analysis

The particle size distribution and surface zeta potential of AZEVs were analyzed using dynamic light scattering (DLS) and electrophoretic light scattering with a Zetasizer Nano ZS90 (Malvern Instruments, UK). For DLS measurements, the detection angle was set to 90°, and the measurement temperature was 25°C. The viscosity and refractive index of the dispersant were set to 0.8872 mPa·s and 1.330, respectively. Samples were suspended in double‐distilled water to approximately 0.5–1 mL and loaded into disposable cuvettes. Measurements were performed using a DTD0012 cuvette with automatic scanning to obtain particle size distribution profiles. For zeta potential analysis, measurements were conducted at 25°C using DTS1070 folded capillary cells. The sample solution was adjusted to fully cover the electrodes to ensure proper conductivity. The dielectric constant and viscosity of the dispersant (water) were set to 78.5 and 0.8872 mPa·s, respectively, and measurements were carried out in automatic mode. The zeta potential was calculated from electrophoretic mobility using the Smoluchowski model and used to evaluate the colloidal stability and aggregation tendency of the vesicles. All measurements were performed in triplicate, and the average particle size distribution and zeta potential were reported.

### 2.5 Transmission Electron Microscopy (TEM) Analysis

The morphology of AZEVs was examined using transmission electron microscopy (TEM). Samples were further purified using a 100 kDa molecular weight cut‐off (MWCO) membrane and concentrated to a final volume of 100 μL by centrifugation at 1000 × *g* for 10 min at 4°C. A 10 μL aliquot of the concentrated sample was applied onto a copper grid and allowed to stand for 20 min before excess liquid was removed with filter paper. The grid was then fixed with 10 μL of 1% paraformaldehyde (PFA) for 15 min, followed by removal of excess fixative. Negative staining was performed using 10 μL of 1% (w/v) uranyl acetate solution; after excess stain was removed, the grid was rinsed once with 10 μL of double‐distilled water and allowed to air dry. Dried grids were kept overnight prior to imaging. TEM observation was performed using a JEM‐1400 Flash transmission electron microscope (JEOL Ltd., Tokyo, Japan) at 80 kV, and images were captured at various magnifications to assess vesicle size and morphology. All TEM operations were conducted with the assistance of the Instrumentation Center at National Chung Hsing University.

### 2.6 Small RNA Sequencing Analysis

Small RNA sequencing and bioinformatics analyses were performed by BIOTOOLS Co. Ltd. (Taiwan). Briefly, small RNA libraries were constructed by sequentially ligating 3′ and 5′ adaptors to small RNAs, followed by reverse transcription and PCR amplification. Libraries containing 18–40 bp inserts were purified and sequenced on an Illumina platform (SE50). Raw image data generated from high‐throughput sequencing were converted into sequencing reads using the CASAVA pipeline and stored in FASTQ format containing sequence information and corresponding quality scores. Low‐quality reads, adaptor sequences, and reads outside the expected size range were removed to obtain clean reads, and the length distribution of small RNAs was analyzed. Clean reads were mapped to the reference genome using Bowtie to determine their genomic distribution and expression levels. Known miRNAs were identified by alignment with sequences deposited in the miRBase database, while novel miRNAs were predicted based on sequence characteristics and secondary structure features. For each identified miRNA, sequence length, read counts, and nucleotide bias at the first position were analyzed.

### 2.7 Cell Culture

HaCaT cells, an immortalized human keratinocyte cell line, were kindly provided by Dr. P. S. Lai (National Chung Hsing University, Taichung, Taiwan). The HaCaT cell line (CVCL_0038) was obtained from AddexBio (Catalog #: T0020001, San Diego, CA, USA). Cells were cultured in high‐glucose Dulbecco's Modified Eagle Medium (DMEM; Gibco, USA; 12100‐046) supplemented with 10% (v/v) fetal bovine serum (FBS), 1% (v/v) penicillin‐streptomycin (Gibco, USA; 15140‐122), and 3.7 g/L sodium bicarbonate (NaHCO_33_). The cells were maintained in a humidified incubator at 37°C with 5% CO_2_.

### 2.8 Cell Scratch Test

For the wound healing assay, HaCaT cells were seeded at a density of 1 × 10^6^ cells per well in 6‐well plates and incubated for 24 h to allow monolayer formation. A scratch was created across the cell monolayer using a sterile pipette tip, and detached cells and debris were removed by washing with PBS. Cells were then divided into a control group (untreated) and treatment groups receiving different concentrations of AZEVs. Images of the wound area were captured at 0, 12, and 24 h using an upright fluorescence microscope (ECLIPSE Ci, Nikon, Japan). Wound closure was quantified with ImageJ software, and the percentage of wound healing was calculated relative to the initial wound area at 0 h.

### 2.9 UVB Irradiation and Drug Treatment

HaCaT cells were seeded at a density of 2 × 10^4^ cells per well in 96‐well plates and incubated for 24 h. After incubation, cells were pretreated with the test compounds (AZEVs and resveratrol as a positive control) dissolved in culture medium containing 0.1% DMSO for 6 h. Following pretreatment, the cells were washed with 1× PBS and irradiated with UVB at 150 mJ/cm^2^ using a UV crosslinker (CL‐3000, Analytik Jena, Germany) in PBS. After UVB exposure, PBS was replaced with fresh culture medium, and the cells were further incubated for 24 h before subsequent assays.

### 2.10 Cell Viability Assay

Cell viability was assessed using the Cell Counting Kit‐8 (CCK‐8; WST‐8), which contains the water‐soluble tetrazolium salt WST‐8 [2‐(2‐methoxy‐4‐nitrophenyl)‐3‐(4‐nitrophenyl)‐5‐(2,4‐disulfophenyl)‐2H‐tetrazolium, monosodium salt]. In viable cells, WST‐8 is reduced by cellular dehydrogenases to produce an orange‐colored formazan dye. The intensity of the color, measured by absorbance at 450 nm, is directly proportional to the number of living cells. For cell viability assays, the culture medium was replaced with fresh medium containing 1% (v/v) Enhanced Cell Counting Kit‐8 reagent. Cells were then incubated at 37°C for 1 h, and absorbance at 450 nm was measured using a microplate spectrophotometer (μQuant, BioTek, USA) to determine cell viability.

$$\text{Cell viability}\;\left(\%\right)=\left(\text{Treatment}\;{\mathrm{OD}}_{450}/\text{Control}\;{\mathrm{OD}}_{450}\right)\times 100.$$

### 2.11 Intracellular Reactive Oxygen Species (ROS) Assay

Intracellular reactive oxygen species (ROS) accumulation in HaCaT cells was assessed using the fluorescent probe 2′,7′‐dichlorofluorescin diacetate (H_2_DCF‐DA), with minor modifications based on previously reported methods [24]. For intracellular ROS assays, the medium was removed, and the cells were washed twice with 1× PBS. H_2_DCF‐DA was dissolved in PBS to a final concentration of 30 μM and added to each well. After incubation for 30 min at 37°C in the dark, fluorescence intensity was measured using a multilabel microplate reader (Hidex, Finland) at excitation/emission wavelengths of 485/535 nm. The percentage of ROS generation was calculated using the following formula:

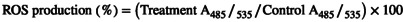




### 2.12 ATP Assay

The intracellular ATP content of HaCaT cells was determined using a luminescence‐based ATP assay, following the method described by Li et al. [25]. ATP levels were measured using an ATP detection assay kit‐luminescence (Cayman Chemical, USA) according to the manufacturer's instructions. Luminescence was detected at 535 nm using a multilabel microplate reader (Hidex, Finland). ATP concentrations were calculated based on standard curves and normalized to the control group, which was set as 100%.

### 2.13 Mitochondrial Membrane Potential Assay

Mitochondrial function in differentiated HaCaT cells was assessed by measuring the mitochondrial membrane potential (Δψm) using JC‐1 dye (5,5′,6,6′‐tetrachloro‐1,1′,3,3′‐tetraethylbenzimidazolylcarbocyanine iodide), a cationic fluorescent probe that accumulates in mitochondria in a membrane potential‐dependent manner. The experimental procedure was adapted from a previously described method [26]. Mitochondrial membrane potential was evaluated using the JC‐1 mitochondrial membrane potential assay kit (Abbkine, China; KTA4001) following the manufacturer's protocol. Briefly, the JC‐1 staining solution was freshly prepared at the recommended working concentration (20 μL JC‐1 dye per 1 mL assay buffer). Cells were incubated with the staining solution at 37°C for 30 min in the dark. After incubation, cells were washed with PBS and subsequently imaged in PBS. Fluorescence imaging was performed with an upright fluorescence microscope (ECLIPSE Ci, Nikon, Japan) equipped with a 40× objective lens. Images were captured using a microscopic imaging system (SGHD‐3.6, SAGE Vision, Taiwan) connected to SG Image V2.3 software (Taiwan). Quantification of fluorescence intensity and channel merging were performed using ImageJ software (National Institutes of Health, USA). Mitochondrial membrane potential (Δψm) was quantified by calculating the ratio of red fluorescence intensity (JC‐1 aggregates) to green fluorescence intensity (JC‐1 monomers). A higher red/green fluorescence ratio indicates a higher mitochondrial membrane potential, whereas a lower ratio reflects mitochondrial depolarization. The calculated red/green fluorescence ratio was used for statistical analysis of Δψm.

### 2.14 Western Blot Analysis

Western blotting was conducted to evaluate the expression of mitochondrial biogenesis and tight junction proteins in HaCaT cells, with modifications based on previously described protocols [4, 27].

For protein extraction, cells were washed with cold PBS, scraped, and lysed in RIPA buffer (Pierce, Thermo Fisher Scientific, USA) supplemented with 1% protease and phosphatase inhibitor cocktails. Lysates were incubated on ice with intermittent vortexing, then centrifuged at high speed. Supernatants were collected and stored at −80°C. Protein concentrations were determined using the BCA assay and adjusted to 90 μg/mL. Samples were denatured at 37°C for 5 min prior to electrophoresis.

Proteins were separated on 12% SDS‐PAGE gels and transferred to PVDF membranes (Revvity, USA) at 300 mA for 1 h. Membranes were washed with TBST and blocked with EZBlocker (GenePure, Taiwan) for 90 min at room temperature. Membranes were then incubated overnight at 4°C with primary antibodies targeting: occludin (1:10,000; 27260‐1‐AP, Proteintech, China), TFAM (1:20,000; 22586‐1‐AP, Proteintech, China), PGC‐1*α* (1:10,000; 66369‐1‐lg, Proteintech, China), ZO‐1 (1:10,000; 21773‐1‐AP, Proteintech, China), claudin‐3 (1:1000; 34‐1700, Invitrogen, USA), AMPK*α*1 (1:1000; 07‐350, Millipore Sigma, USA), *p*‐AMPK*α* (1:1000; 2535, Cell Signaling Technology, USA), SIRT‐1 (1:1000; 9475s, Cell Signaling Technology, USA), Nrf2 (1:1000; 12721, Cell Signaling Technology, USA), *β*‐actin (1:5000; SC‐47778, Santa Cruz, UK).

Following primary antibody incubation, membranes were washed with TBST and incubated with species‐specific HRP‐conjugated secondary antibodies: goat anti‐mouse IgG (1:10,000; ARG65350, Arigo Biolaboratories, China) or rabbit IgG (1:10,000; abs‐22‐200, Asia Bioscience, Taiwan) for 2 h at room temperature with gentle shaking. After final washes with TBST, immunoreactive bands were visualized using WesternBright ECL substrate (Advansta, USA) and detected via chemiluminescence.

### 2.15 Statistical Analysis

All data are presented as mean ± standard deviation (SD) from at least three independent experiments (*n* ≥ 3), unless otherwise specified. The exact *n* values for each experiment are indicated in the corresponding figure legends. Statistical analyses were performed using GraphPad Prism 8.0 software (Dotmatics, USA). Comparisons among multiple groups were evaluated using one‐way analysis of variance (ANOVA) followed by Tukey's honestly significant difference (HSD) post hoc test. A *p*‐value < 0.05 was considered statistically significant.

## 3 Result

### 3.1 Characterization of *Alpinia zerumbet* ‐Derived Extracellular Vesicles (AZEVs)

**FIGURE 1:**
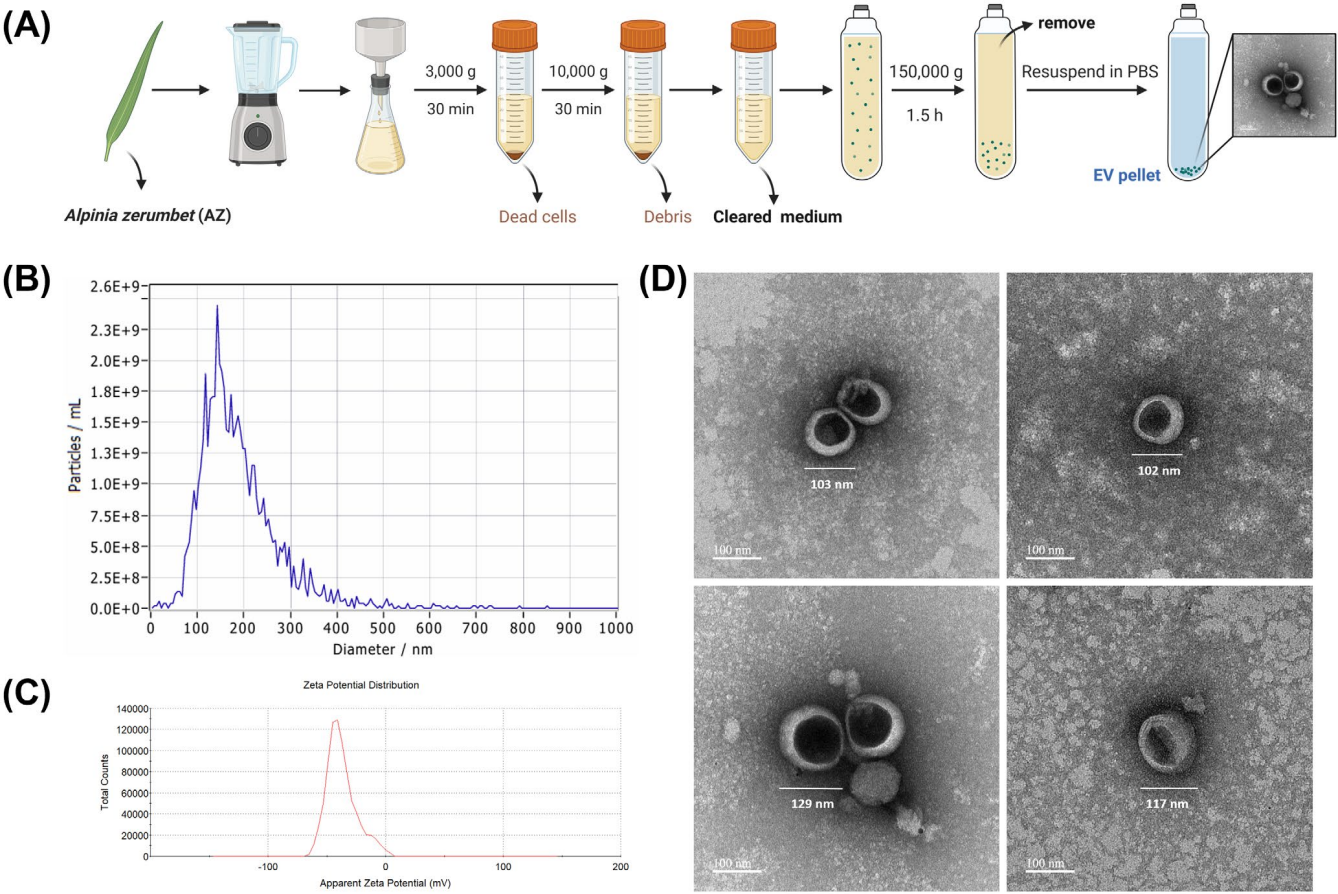
Isolation and characterization of extracellular vesicles derived from *Alpinia zerumbet* (AZEVs). (A) Schematic illustration of the differential ultracentrifugation workflow used to isolate AZ‐EVs. Plant tissues were homogenized and sequentially centrifuged (3000 × *g* and 10,000 × *g*) to remove dead cells and debris, followed by ultracentrifugation at 150,000 × *g* to collect EV pellets, which were then resuspended in PBS. (B) Nanoparticle tracking analysis (NTA) showing the size distribution and particle concentration of AZEVs. (C) Zeta potential distribution indicating the surface charge characteristics of the isolated vesicles. (D) Transmission electron microscopy (TEM) images demonstrating the typical spherical or cup‐shaped morphology of AZEVs with diameters around 100–130 nm. Scale bar = 100 nm.

Extracellular vesicles derived from *Alpinia zerumbet* leaves (AZEVs) were isolated using differential ultracentrifugation (Figure 1A). Fresh leaves were homogenized, followed by sequential centrifugation to remove cell debris and large particles, and finally ultracentrifuged at 150,000 × *g* to pellet the extracellular vesicles. The resulting pellet was resuspended in double‐distilled water for subsequent characterization. Nanoparticle tracking analysis (NTA) revealed that the isolated vesicles exhibited a size distribution primarily between 100 and 300 nm, with an average diameter of 150.8 ± 4.2 nm and a concentration of 8.55 ± 9.37 × 10^13^ particles/mL (Figure 1B), consistent with previously reported size ranges of plant‐derived extracellular vesicles. Zeta potential analysis further indicated that AZEVs carried a negative surface charge, with an average zeta potential of −36.4 ± 6.0 mV and a polydispersity index (PDI) of 0.152 ± 0.019 (Figure 1C). The negative surface charge is likely attributable to anionic membrane components, such as phospholipids, glycolipids, and surface‐associated biomolecules, which are commonly found in plant‐derived extracellular vesicles. Such electrostatic properties may contribute to the observed colloidal stability by preventing vesicle aggregation through electrostatic repulsion, suggesting good stability in aqueous suspension. Transmission electron microscopy (TEM) revealed that AZEVs exhibited typical spherical to cup‐shaped morphology with a clear lipid bilayer structure (Figure 1D). In addition, ^1^H‐NMR analysis was performed as a supplementary chemical characterization, showing broad signals consistent with carbohydrate‐ and lipid‐associated components (Figure S1), further supporting the heterogeneous biomolecular composition of AZEVs. Observed vesicle diameters ranged from approximately 100–150 nm, consistent with the NTA measurements. Collectively, these results confirm the successful isolation of nanosized extracellular vesicles from *A. zerumbet* leaves, exhibiting characteristic EV size distribution, negative surface charge, and membrane‐bound morphology.

### 3.2 AZEVs Characterization and Composition Identification of Small RNAs (sRNAs) in AZEVs

**FIGURE 2:**
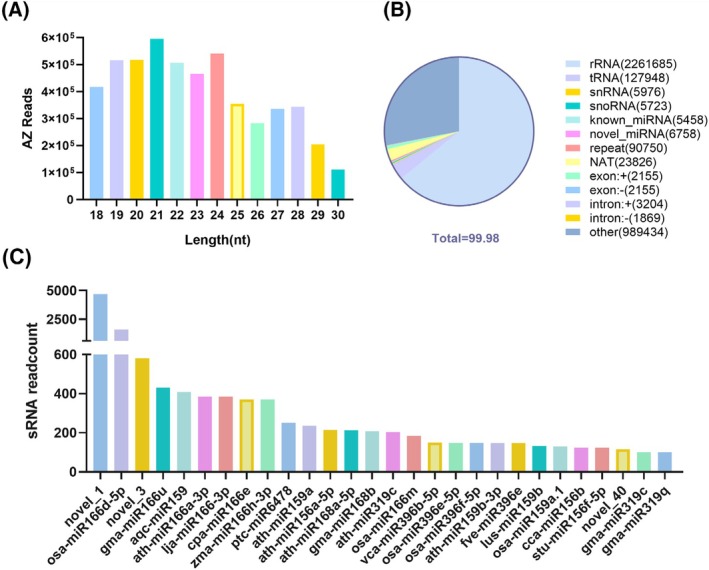
Small RNA sequencing analysis of extracellular vesicles derived from *Alpinia zerumbet* leaves (AZEVs). (A) Length distribution of small RNA reads in AZEVs, showing that most sequences ranged from 18 to 30 nt, with a predominant peak around 20–24 nt. (B) Classification of small RNA categories identified from sequencing data, including rRNA, tRNA, snRNA, snoRNA, known miRNA, novel miRNA, repeat‐associated RNAs, NAT, exon‐ and intron‐derived RNAs, and other small RNA species. (C) Abundance distribution of the top identified miRNAs in AZEVs based on sequencing read counts.

To further investigate the molecular mechanisms underlying the bioactivity of AZEVs, we performed small RNA sequencing to profile the RNA cargo within the vesicles. Analysis of read length distribution revealed that the majority of sRNAs in AZEVs ranged from 18 to 24 nucleotides (nt), with a predominant peak at 21 nt, consistent with the canonical size distribution of plant microRNAs (miRNAs) and small interfering RNAs (siRNAs) (Figure 2A). Library composition analysis indicated that AZEVs contained diverse RNA species, including rRNA (2,261,685 reads), tRNA, snRNA, and snoRNA (Figure 2B). Notably, we identified 5458 known miRNAs and 6758 predicted novel miRNAs, demonstrating that AZEVs carry a highly heterogeneous population of regulatory RNAs. Abundance analysis of the most highly expressed sequences revealed that the top‐ranking miRNA was novel_1 with nearly 5000 reads, followed by osa‐miR166d‐5p (Figure 2C). Several highly conserved plant miRNA families involved in growth and stress responses, including miR166, miR159, miR156, and miR396, were also abundantly present in AZEVs. Detailed sequences and corresponding read counts of these major miRNAs are summarized in Table 1. Collectively, these results demonstrate that AZEVs are enriched with small RNAs possessing potential bioactivity, particularly members of the miR166 and miR159 families, providing a foundational dataset for exploring their cross‐kingdom regulatory functions, such as anti‐inflammatory or wound‐healing effects.

**TABLE 1:** Sequences of the miRNAs identified through sequencing.

miRNA name	Mature_sequence	Readcount
novel_1	CUAUCUAAGGAACAGUGACUU	4685
osa‐miR166d‐5p	GGAAUGUUGUCUGGCUCGAGG	1597
novel_3	UAUCUAAGGAAUAGUGACUU	580
gma‐miR166u	UCUCGGACCAGGCUUCAUUC	431
aqc‐miR159	UUUGGACUGAAGGGAGCUCUA	409
ath‐miR166a‐3p	UCGGACCAGGCUUCAUUCCCC	384
lja‐miR166‐3p	AUUUCGGACCAGGCUUCAUUCCCC	384
cpa‐miR166e	GGACCAGGCUUCAUUCCCC	371
zma‐miR166h‐3p	UCGGACCAGGCUUCAUUCCC	371
ptc‐miR6478	CCGACCUUAGCUCAGUUGGUG	251
ath‐miR159a	UUUGGAUUGAAGGGAGCUCUA	236
ath‐miR156a‐5p	UGACAGAAGAGAGUGAGCAC	215
ath‐miR168a‐5p	UCGCUUGGUGCAGGUCGGGAA	213
gma‐miR168b	UCGCUUGGUGCAGGUCGGG	208
ath‐miR319c	UUGGACUGAAGGGAGCUCCUU	203
osa‐miR166m	UCGGACCAGGCUUCAUUCCCU	184
vca‐miR396b‐5p	UCCACAGGCUUUCUUGAACUA	150
osa‐miR396e‐5p	UCCACAGGCUUUCUUGAACUG	149
osa‐miR396f‐5p	UCUCCACAGGCUUUCUUGAACU	149
ath‐miR159b‐3p	UUUGGAUUGAAGGGAGCUCUU	147
fve‐miR396e	UUCCACAGGCUUUCUUGAACU	147
lus‐miR159b	UUUGGAUUGAAGGGAGCUCUC	132
osa‐miR159a.1	UUUGGAUUGAAGGGAGCUCUG	131
cca‐miR156b	UGACAGAAGAGAGUGAGCAUA	123
stu‐miR156f‐5p	CUGACAGAAGAGAGUGAGCA	123
novel_40	GUGCAGUUCUCCUUUGGCAGG	116
gma‐miR319c	UUGGACUGAAGGGAGCUCCU	101
gma‐miR319q	UGGACUGAAGGGAGCUCCUUC	101

### 3.3 AZEVs Enhance HaCaT Cell Viability and Promote Wound Healing

**FIGURE 3:**
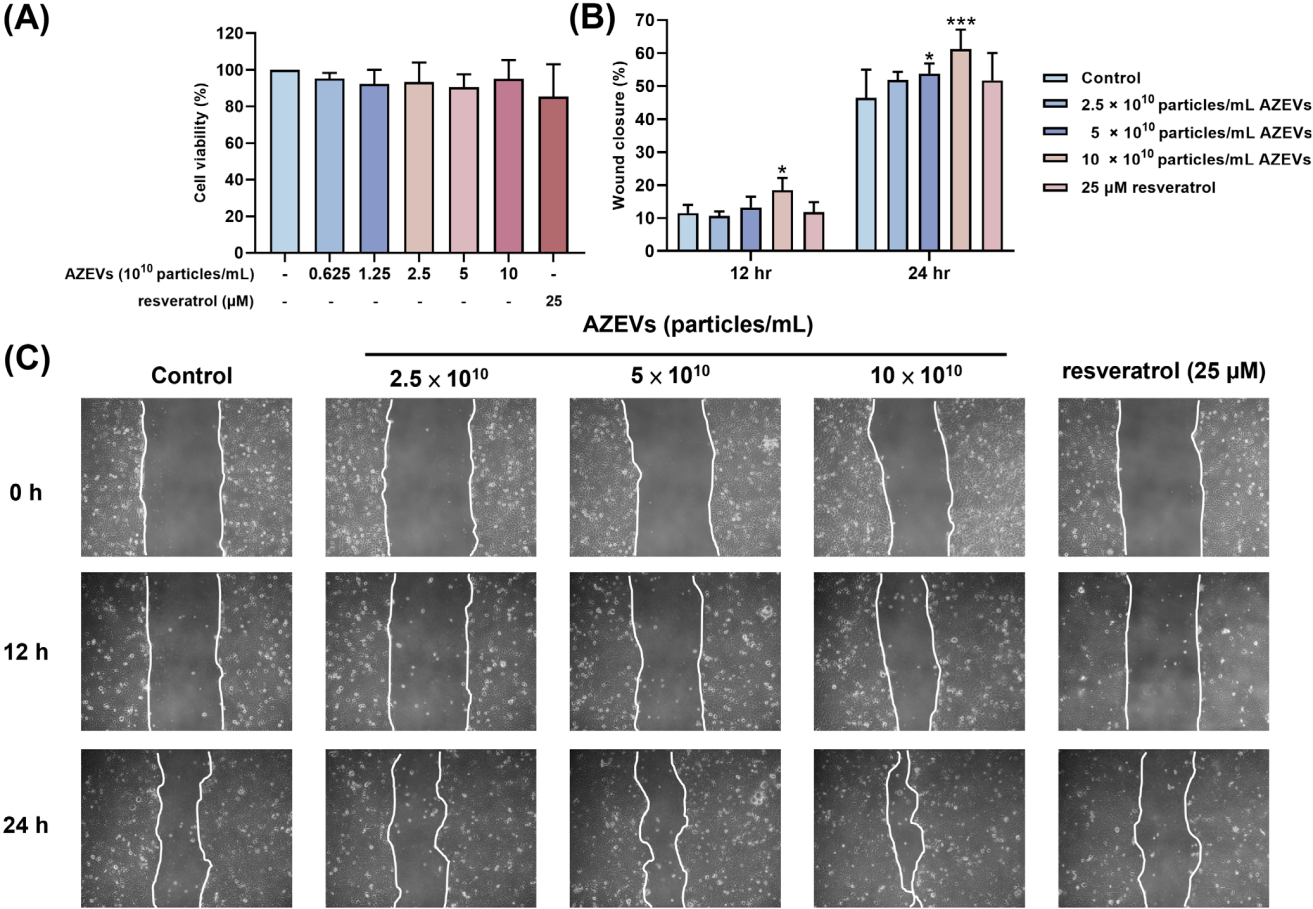
Effects of *Alpinia zerumbet* ‐derived extracellular vesicles (AZEVs) on HaCaT cell viability and migration. (A) Cell viability after treatment with different concentrations of AZEVs and resveratrol. (B) Quantification of wound closure in HaCaT cells treated with AZEVs and resveratrol. Data are presented as the percentage of wound closure relative to the initial wound area. (C) Representative microscopic images of the scratch wound‐healing assay in HaCaT cells treated with AZEVs and resveratrol at 0, 12, and 24 h. Images were captured using a microscope with a 10× objective. Scale bars represent the indicated magnification. Data are presented as mean ± SD (*n* = 3). Statistical analysis was performed using one‐way ANOVA followed by Tukey's HSD post hoc test. *p < 0.05; ***p < 0.001 versus the control group. Resveratrol (25 μM) was used as a positive control.

To evaluate the potential of AZEVs in skin regeneration, we first assessed their effects on the viability and migration of HaCaT cells. As shown in Figure 3A, treatment with AZEVs at concentrations ranging from 0.625 × 10^10^ to 10 × 10^10^ particles/mL for 24 h had no significant effect on cell viability, confirming that AZEVs were non‐cytotoxic at the tested doses. Resveratrol (25 μM), used as a positive control, also did not adversely affect cell proliferation. We then examined the effects of AZEVs on HaCaT cell migration. In the scratch assay, cells treated with AZEVs displayed dose‐dependent wound closure at both 12 and 24 h (Figure 3B,C). Statistical analysis revealed that higher doses (5 × 10^10^ and 10 × 10^10^ particles/mL) significantly enhanced wound closure at 24 h compared with the untreated control. These findings indicate that AZEVs at appropriate concentrations effectively promote HaCaT cell migration, thereby accelerating wound healing.

### 3.4 AZEVs Alleviate UVB‐Induced Cytotoxicity and Improve Mitochondrial Function in HaCaT Keratinocytes

**FIGURE 4:**
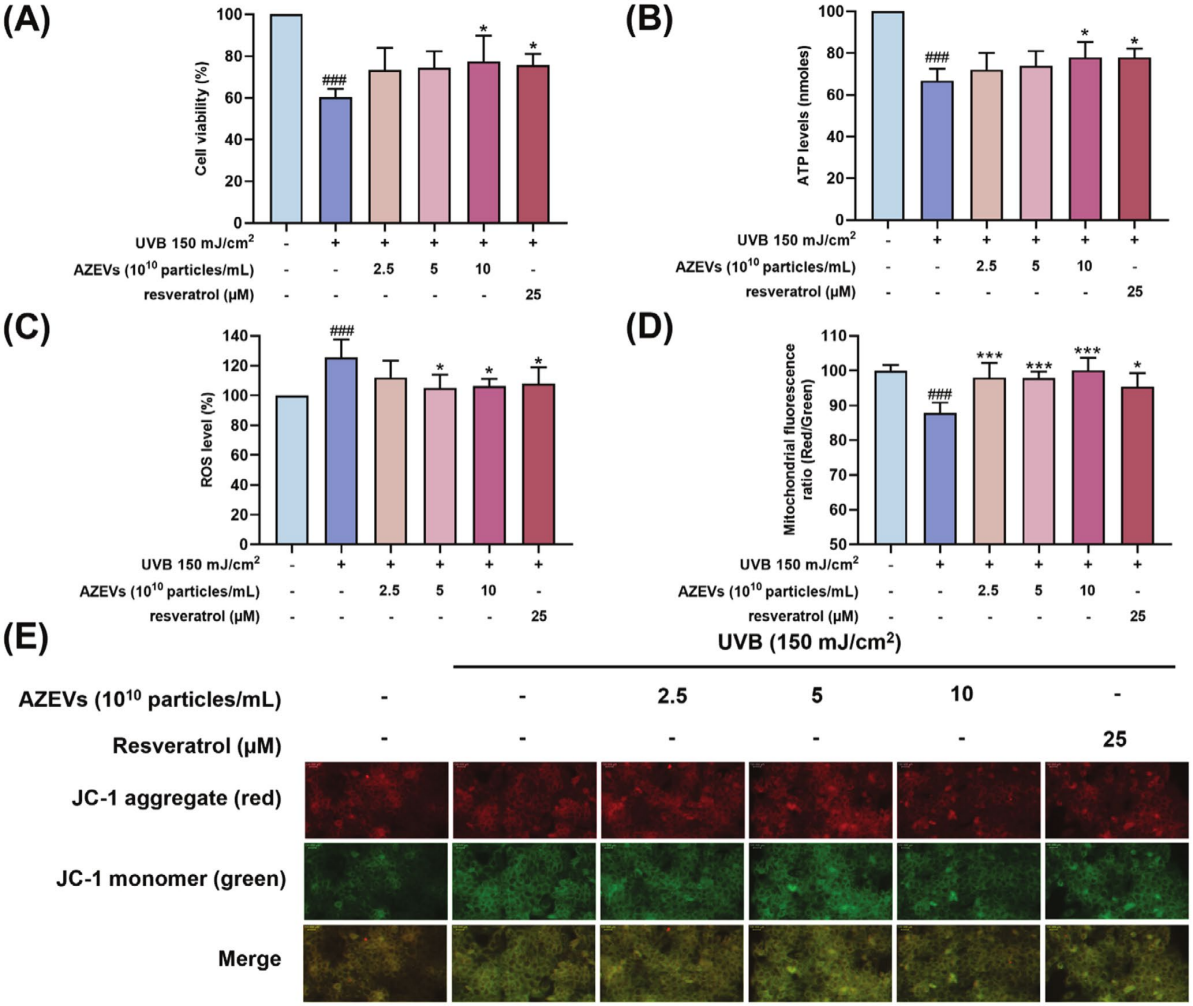
Effects of AZEVs on cell viability, ROS production, and mitochondrial function in UVB‐irradiated HaCaT cells. (A) Cell viability after treatment with different concentrations of AZEVs and resveratrol in UVB‐irradiated HaCaT cells. (B) Intracellular ATP levels. (C) Intracellular ROS levels. (D) Quantitative analysis of mitochondrial membrane potential was performed by calculating the ratio of red fluorescence (JC‐1 aggregates) to green fluorescence (JC‐1 monomers). (E) Representative fluorescence images of mitochondrial membrane potential assessed by JC‐1 staining. Red fluorescence indicates JC‐1 aggregates (healthy mitochondria), and green fluorescence indicates JC‐1 monomers (depolarized mitochondria). Nuclei were stained with DAPI. Images were captured using a fluorescence microscope with a 40× objective. Data are presented as mean ± SD (*n* = 3). Statistical analysis was performed using one‐way ANOVA followed by Tukey's HSD post hoc test. ^###^ *p* < 0.001 versus the control group; *p < 0.05; ***p < 0.001 versus UVB group. Resveratrol (25 μM) was used as a positive control.

To evaluate the protective and restorative effects of AZEVs on HaCaT cells following UVB exposure, cell viability was first assessed using the CCK‐8 assay. Resveratrol, a polyphenolic compound with well‐documented antioxidant and anti‐inflammatory properties, was included as a positive control [28]. The results demonstrated that UVB irradiation (150 mJ/cm^2^) significantly reduced cell viability and intracellular ATP production, while markedly increasing ROS levels, indicating severe UVB‐induced cytotoxicity and oxidative stress (Figure 4A–C). However, treatment with AZEVs (2.5 × 10^10^–10 × 10^10^ particles/mL) led to a dose‐dependent recovery of cell viability and ATP levels, effectively suppressing ROS accumulation and restoring them to near‐control levels. Furthermore, since mitochondrial integrity is crucial for cell survival, we evaluated changes in mitochondrial membrane potential (Δψm) using JC‐1 staining. In healthy mitochondria, JC‐1 forms aggregates that emit red fluorescence; however, in damaged or depolarized mitochondria, it remains as monomers that emit green fluorescence. As shown in Figure 4E, UVB exposure caused a pronounced shift from red to green fluorescence, signifying a significant loss of Δψm. Quantitative analysis confirmed that AZEV treatment effectively increased the red‐to‐green fluorescence ratio, thereby restoring mitochondrial membrane potential (Figure 4D). Taken together, these findings suggest that AZEVs mitigate UVB‐induced cellular damage and mitochondrial dysfunction by inhibiting oxidative stress, maintaining membrane potential, and restoring cellular energy metabolism, highlighting their potential as a restorative agent against photoaging.

### 3.5 AZEVs Improve Mitochondrial Biogenesis‐Related Signaling Pathways in UVB‐Irradiated Keratinocytes

**FIGURE 5:**
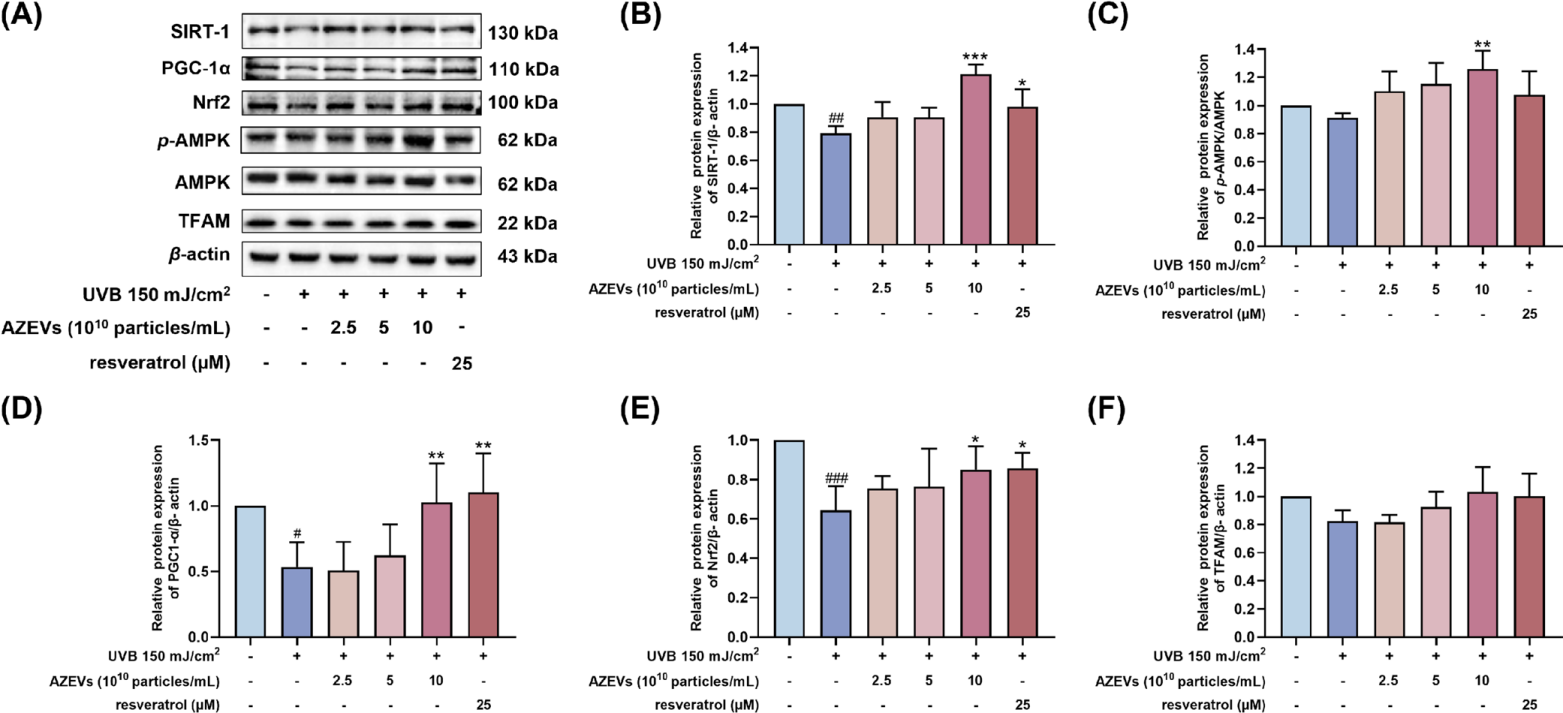
The effects of AZEVs on the expression of mitochondrial biogenesis protein in UVB‐irradiated HaCaT cells. (A) Western blotting visual image of SIRT‐1, PGC‐1*α*, Nrf2, *p*‐AMPK, AMPK, and TFAM. Relative protein expression levels of (B) SIRT‐1, (C) *p*‐AMPK/AMPK, (D) PGC‐1*α*, (E) Nrf2, and (F) TFAM in the HaCaT cells. *β*‐actin was used as the protein loading control. Data are presented as mean ± SD (*n* = 4). Statistical analysis was performed using one‐way ANOVA followed by Tukey's HSD post hoc test. ^#^ *p* < 0.05; ^##^ *p* < 0.01; ^###^ *p* < 0.001 versus the control group; *p < 0.05; **p < 0.01; ***p < 0.001 versus UVB group. Resveratrol (25 μM) was used as a positive control.

To investigate whether AZEVs alleviate UVB‐induced cellular damage by regulating mitochondrial function‐related signaling pathways, the expression levels of key regulatory proteins, including SIRT‐1, PGC‐1*α*, Nrf2, *p*‐AMPK/AMPK, and TFAM, were evaluated. UVB irradiation markedly reduced the expression of SIRT‐1, PGC‐*α*, and Nrf2 compared to the non‐irradiated control group (Figure 5A,B,D,E). Treatment with AZEVs (2.5 × 10^10^–10 × 10^10^ particles/mL) significantly restored the expression of SIRT‐1, PGC‐1*α*, and Nrf2 in a dose‐dependent manner, with prominent effects observed at the highest concentration (10 × 10^10^ particles/mL), which even surpassed the effect of the positive control, resveratrol (Figure 5B,D,E). Notably, AZEV treatment at the highest dose (10 × 10^10^ particles/mL) significantly increased the *p*‐AMPK/AMPK ratio (Figure 5C), suggesting that AZEVs may partially exert their protective effects by activating the AMPK signaling pathway. While TFAM expression showed a trend toward restoration in the AZEV‐treated groups, the difference did not reach statistical significance compared with the UVB‐induced group (Figure 5F). Collectively, these findings indicate that AZEVs counteract UVB‐induced suppression of proteins involved in mitochondrial biogenesis‐related signaling and antioxidant defense, primarily by modulating the SIRT‐1/PGC‐1*α*/Nrf2 axis and activating AMPK.

### 3.6 AZEVs Restore Barrier Function in UVB‐Irradiated Keratinocytes

**FIGURE 6:**
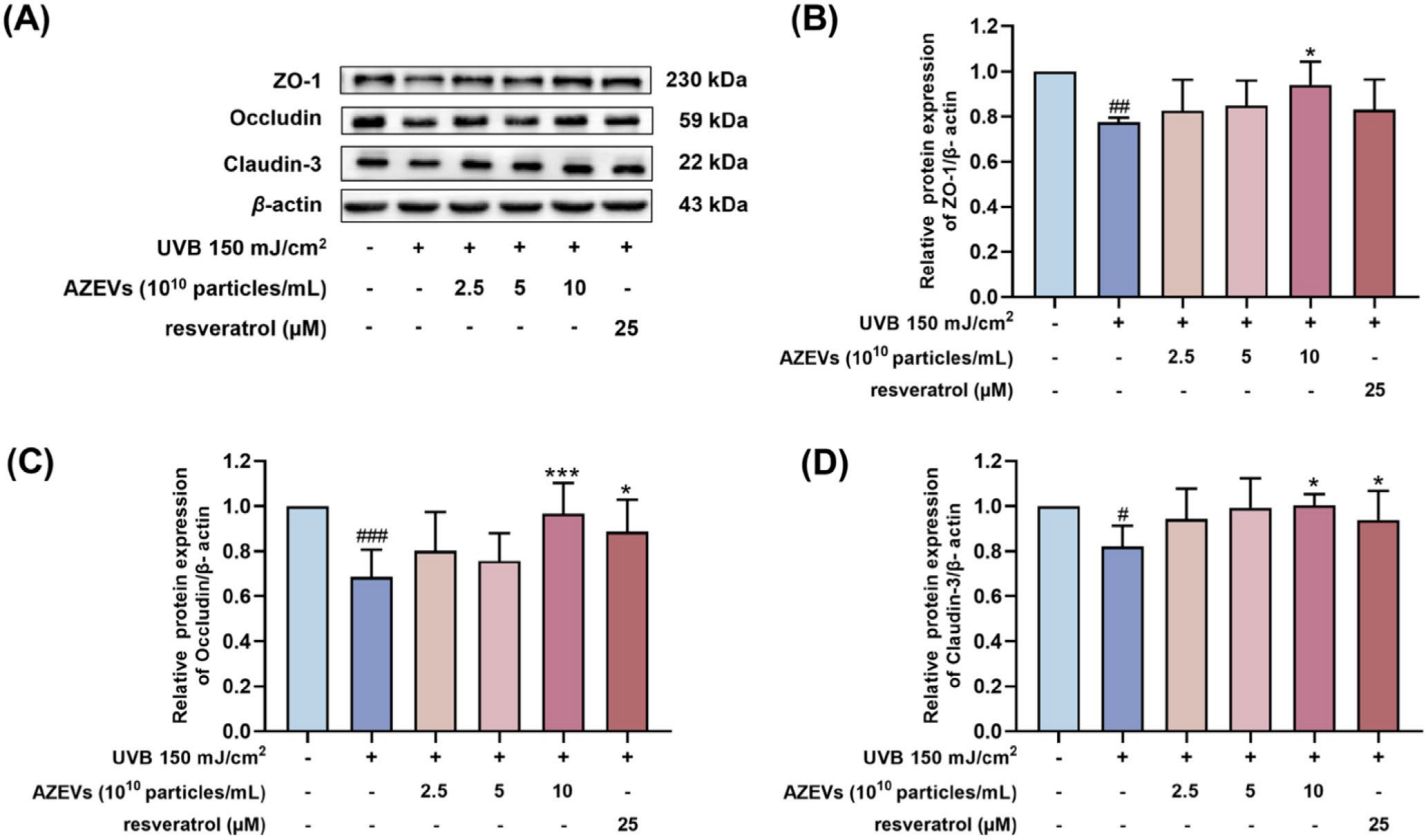
The effects of AZEVs on the expression of tight junction protein in UVB‐irradiated HaCaT cells. (A) Western blotting visual image of ZO‐1, occludin, and claudin‐3. Relative protein expression of (B) ZO‐1, (C) occludin, and (D) claudin‐3 in different groups. *β*‐actin was used as the protein loading control. Data are presented as mean ± SD (*n* = 4). Statistical analysis was performed using one‐way ANOVA followed by Tukey's HSD post hoc test. ^#^ *p* < 0.05; ^##^ *p* < 0.01; ^###^ *p* < 0.001 versus the control group; *p < 0.05, ***p < 0.001 versus UVB group. Resveratrol (25 μM) was used as a positive control.

To investigate whether AZEVs can alleviate UVB‐induced skin barrier damage, this study further examined the expression of tight junction proteins, including ZO‐1, occludin, and claudin‐3. As shown in Figure 6A, UVB irradiation significantly decreased the protein expression of ZO‐1, occludin, and claudin‐3. However, treatment with AZEVs restored the expression of these tight junction proteins in a dose‐dependent manner (Figure 6B–D). In conclusion, these results suggest that AZEVs effectively maintain tight junction protein expression under UVB irradiation, thereby preserving skin barrier integrity. AZEVs may thus be a potential candidate for preventing or repairing UVB‐induced skin damage.

## 4 Discussion

In recent years, plant‐derived extracellular vesicles (PDEVs) have emerged as a class of naturally occurring bioactive nanovesicles capable of mediating cross‐kingdom molecular communication. These vesicles possess a lipid bilayer structure and can carry a diverse range of bioactive molecules, including proteins, lipids, metabolites, and small RNAs, and have been shown to be internalized by mammalian cells, thereby modulating signaling pathways related to inflammation, oxidative stress, and tissue repair [29]. Due to their favorable biocompatibility and natural origin, PDEVs have attracted increasing attention in dermatology and cosmetic applications. *Alpinia zerumbet* , a member of the Zingiberaceae family, has long been recognized for its medicinal and functional properties, as it is rich in polyphenols, flavonoids, and terpenoids. Previous studies have demonstrated its antioxidant, anti‐inflammatory, melanogenesis‐inhibitory, and anti‐photoaging effects, suggesting potential applications in skin health [23, 30, 31]. Recent research further indicates that PDEVs contain various bioactive compounds from their parent plants, including saponins, polyphenols, flavonoids, glucuronides, terpenoids, and carotenoids, which retain their biological activities even after traversing physiological barriers [32]. These observations suggest that part of the bioactivity of plants may be mediated by their released EVs, which serve as natural carriers to deliver active molecules or regulatory RNAs to recipient cells and modulate associated physiological responses. Moreover, owing to their lipid bilayer‐mediated membrane fusion properties, plant EVs can penetrate the stratum corneum and even reach deeper skin layers via follicular pathways, thereby potentially exerting protective and restorative effects against photoaging [33]. In this study, we successfully isolated extracellular vesicles from *A. zerumbet* leaves (AZEVs). Characterization by nanoparticle tracking analysis, zeta potential measurement, and transmission electron microscopy confirmed their nanoscale size, negative surface charge, and lipid bilayer morphology, consistent with typical plant EV features. The ^1^H‐NMR spectra of AZEVs showed prominent carbohydrate‐associated signals, whereas lipid‐related resonances appeared as broad, unresolved peaks. This is consistent with the inherent limitations of NMR analysis in complex extracellular vesicle mixtures, where overlapping signals from heterogeneous lipid and protein components often preclude detailed structural resolution. Therefore, the NMR results should be interpreted as supportive evidence rather than definitive structural identification. These findings establish AZEVs as stable nanovesicles and provide a mechanistic basis for understanding the bioactive potential of *A. zerumbet* in skin applications.

Mitochondria play a central role in maintaining energy metabolism and redox homeostasis in skin cells. UVB irradiation is known to impair mitochondrial function, leading to decreased ATP production, increased ROS accumulation, and reduced mitochondrial membrane potential, ultimately resulting in cellular damage and photoaging [34]. The integrity of the skin barrier is highly dependent on cellular energy status, as keratinocyte differentiation, tight junction formation, and epidermal turnover all require sufficient ATP supply [35, 36]. Consistent with this, our results demonstrate that AZEVs significantly restored ATP levels, reduced ROS generation, and improved mitochondrial membrane potential in UVB‐exposed HaCaT cells, indicating a protective effect on mitochondrial function. Mechanistically, AZEVs upregulated key regulators of mitochondrial biogenesis‐related pathway protein, including SIRT‐1, PGC‐1*α*, and Nrf2, and activated AMPK signaling. The SIRT‐1/PGC‐1*α* axis is critical for controlling mitochondrial biogenesis and energy metabolism, whereas Nrf2 mediates cellular antioxidant defense [6]. These findings suggest that AZEVs improve mitochondrial function and strengthen cellular resistance to oxidative stress by activating these signaling pathways. Although intracellular ATP levels were restored following AZEV treatment, ATP measurements represent a steady‐state endpoint and do not distinguish ATP derived from oxidative phosphorylation and glycolysis. Therefore, the observed ATP recovery should be interpreted primarily as restoration of cellular energy status rather than direct evidence of enhanced mitochondrial respiration. Future studies employing metabolic flux analysis will be required to further characterize the effects of AZEVs on mitochondrial bioenergetics. Moreover, AZEVs restored the expression of essential tight junction proteins, including ZO‐1, occludin, and claudin‐3, in UVB‐damaged keratinocytes, indicating that improved mitochondrial function may indirectly stabilize and repair epidermal barrier structures. Collectively, these results reinforce the physiological link between mitochondrial function, energy metabolism, and skin barrier integrity, highlighting AZEVs as a potential natural intervention to mitigate UVB‐induced skin damage.

Small RNA sequencing analysis revealed that AZEVs are enriched in 18–24 nt small RNAs, with a predominant peak at 21 nt, consistent with the typical size distribution of plant miRNAs and siRNAs. Conserved miRNA families such as miR166, miR159, miR156, and miR396, which are known to regulate plant development and stress responses [37], were abundantly present in AZEVs. Although the concept of cross‐kingdom regulation by plant‐derived miRNAs remains debated, with conflicting reports regarding their stability and functionality in mammalian systems [38], these miRNAs may potentially modulate cellular pathways such as inflammation and metabolism. Notably, the most abundant sequence detected, novel_1 (CUAUCUAAGGAACAGUGACUU, 21 nt), exhibits a 7‐nt seed region (UAUCUAA) with complementary motifs (AUAGAUU) prevalent in the 3′UTRs of numerous human mRNAs. Target prediction using miRanda (https://www.microrna.org) identified high‐confidence potential targets, including SLC7A11, USP4, AZIN1, and PPP4C, which are involved in redox balance, mitochondrial function, and cellular energy metabolism. These predicted targets align with our functional observations in HaCaT cells, where AZEVs enhanced ATP production, reduced ROS accumulation, and restored mitochondrial membrane potential, suggesting that novel_1 may contribute to mitochondrial function and oxidative stress regulation. While direct regulation of tight junction proteins (e.g., ZO‐1, occludin, claudin‐3) by novel_1 has not been demonstrated, its effects on mitochondrial function and ROS homeostasis may indirectly support epidermal barrier repair, consistent with the observed recovery of tight junction protein expression in UVB‐damaged cells. For example, SLC7A11 promotes cysteine uptake and glutathione synthesis, reducing intracellular oxidative stress [38]; USP4 stabilizes Nrf2 via deubiquitination, enhancing antioxidant responses [39]; and PPP4C may regulate AMPK signaling [40]. Overall, these molecular insights correspond with the activation of SIRT1/PGC‐1*α*/Nrf2/AMPK signaling observed experimentally, supporting a potential miRNA‐mediated mechanism by which AZEVs promote mitochondrial biogenesis‐related pathways and maintain redox homeostasis in skin cells.

Although cross‐kingdom regulation by plant miRNAs remains controversial, and the functional role of the identified novel miRNA (novel_1) requires further validation, for example via target verification using luciferase reporter assays or AGO‐RIP, our results provide a plausible hypothesis that AZEVs may act as natural molecular carriers, delivering functional small RNAs to mammalian skin cells to mediate antioxidant activity, mitochondrial protection, and maintenance of epidermal barrier integrity. Despite the observed protective effects of AZEVs against UVB‐induced skin cell damage, several limitations remain. First, the stability and functional relevance of plant‐derived miRNAs in mammalian cells require further investigation. Second, the targets of novel_1 have not yet been experimentally confirmed; future studies integrating transcriptomic profiling, gene knockdown, or overexpression approaches could clarify the role of AZEV small RNAs in skin protection. Moreover, translation of AZEVs faces challenges in production and scale‐up, including the need for standardized, GMP‐compliant manufacturing and purification methods, such as ultrafiltration, size‐exclusion chromatography, or affinity‐based isolation, to ensure vesicle purity and integrity. Finally, although PDEVs are generally considered biocompatible, their safety and long‐term tolerability require systematic evaluation through toxicology, immunogenicity, and chronic skin exposure studies. Overall, this study provides a first mechanistic insight into the potential role of *Alpinia zerumbet* ‐derived EVs in mitigating photoaging through improvements in mitochondrial function and small RNA regulation, highlighting their promise as natural nanocarriers for anti‐aging and skin repair applications.

## 5 Conclusion

In this study, we successfully isolated and characterized extracellular vesicles derived from *Alpinia zerumbet* leaves (AZEVs), confirming their stability and typical EV features. Functional analyses demonstrated that AZEVs effectively alleviated UVB‐induced damage in HaCaT keratinocytes by ROS, restoring mitochondrial membrane potential and enhancing ATP production, thereby providing essential energy support for the expression of tight junction proteins (ZO‐1, occludin, and claudin‐3) and maintaining epidermal barrier integrity. Moreover, AZEVs upregulated SIRT1, PGC‐1*α*, Nrf2, and AMPK signaling pathways, indicating promotion of mitochondrial biogenesis‐related pathways and enhancement of cellular antioxidant defense mechanisms. AZEVs also facilitated HaCaT cell migration and accelerated wound closure, highlighting their potential in skin repair and regeneration. Small RNA sequencing revealed that AZEVs were enriched for multiple conserved miRNAs, including a novel miRNA (novel_1), with predicted targets associated with redox balance and energy metabolism, suggesting a possible role in modulating mitochondrial function and cellular defense via miRNA‐mediated regulatory networks. In summary, AZEVs exhibit multifaceted bioactivities, including antioxidant effects, improvement of mitochondrial function, regulation of mitochondrial biogenesis‐related signaling pathways, and facilitation of wound healing, suggesting their potential as natural bioactive agents for skin protection, repair, and anti‐photoaging applications.

## Author Contributions

**Mo‐Rong Xu:** data curation, formal analysis, methodology, writing – original draft. **Hui‐Chun Wang:** investigation, methodology. **Meng‐Shiou Lee:** conceptualization, supervision. **Sheng‐Yang Wang:** investigation, resources, writing – review and editing.

## Funding

This work was supported by grants from the Ministry of Science and Technology, Taiwan (NSTC 112‐2313‐B‐005‐018‐MY3), and China Medical University, Taiwan (CMU114‐MF‐10, CMU114‐S‐12).

## Conflicts of Interest

The authors declare no conflicts of interest.

## Supporting information


**Figure S1:** ^1^H‐NMR spectrum of AZEVs.
The spectrum shows broad carbohydrate‐associated resonances (3–5 ppm) and weak, unresolved aliphatic signals, consistent with lipid‐ and glycan‐associated components typically observed in heterogeneous extracellular vesicle preparations.

## Data Availability

The data that support the findings of this study are available on request from the corresponding author. The data are not publicly available due to privacy or ethical restrictions.
