# Supplementary material for: Alpinia zerumbet‐Derived Extracellular Vesicles Protect Against UVB‐Induced Photoaging by Enhancing Mitochondrial Bioenergetics and Skin Barrier Function
Source: Biofactors. 2026 Jul 3;52(4):e70134. doi: 10.1002/biof.70134 (PMC13331998; doi:10.1002/biof.70134)
Supplement: Supplementary file 1 — Figure S1: 1H‐NMR spectrum of AZEVs. The spectrum shows broad carbohydrate‐associated resonances (3–5 ppm) and weak, unresolved aliphatic signals, consistent with lipid‐ and glycan‐associated components typically observed in heterogeneous extracellular vesicle preparations. [file BIOF-52-0-s001.docx]

***Alpinia zerumbet*-derived extracellular vesicles protect against UVB-induced photoaging by enhancing mitochondrial bioenergetics and skin barrier function**

**Mo-Rong Xu^a^, Hui-Chun Wang^b^, Meng-Shiou Lee^c^, Sheng-Yang Wang^a,d,e*^**

^a^Department of Forestry, National Chung-Hsing University, Taichung 402, Taiwan

^b^Graduate Institute of Natural Products, Kaohsiung Medical University, Kaohsiung 807, Taiwan

^c^Department of Chinese Pharmaceutical Science and Chinese Medicine Resources, China Medical University, Taichung 40402, Taiwan

^d^Program in Specialty Crops and Metabolomics, Academy of Circular Economy, National Chung Hsing University, Nantou 540, Taiwan

^e^Agricultural Biotechnology Research Center, Academia Sinica, Taipei 108, Taiwan

*Correspondence

Sheng-Yang Wang,

Department of Forestry, National Chung-Hsing University, 250 Kuo-Kuang Road, Taichung-402, Taiwan

Fax: +886-4-22873628; Phone: +886-4-22840345 ext. 138.

E-mail: [taiwanfir@dragon.nchu.edu.tw](mailto:taiwanfir@dragon.nchu.edu.tw) (S-Y. Wang)


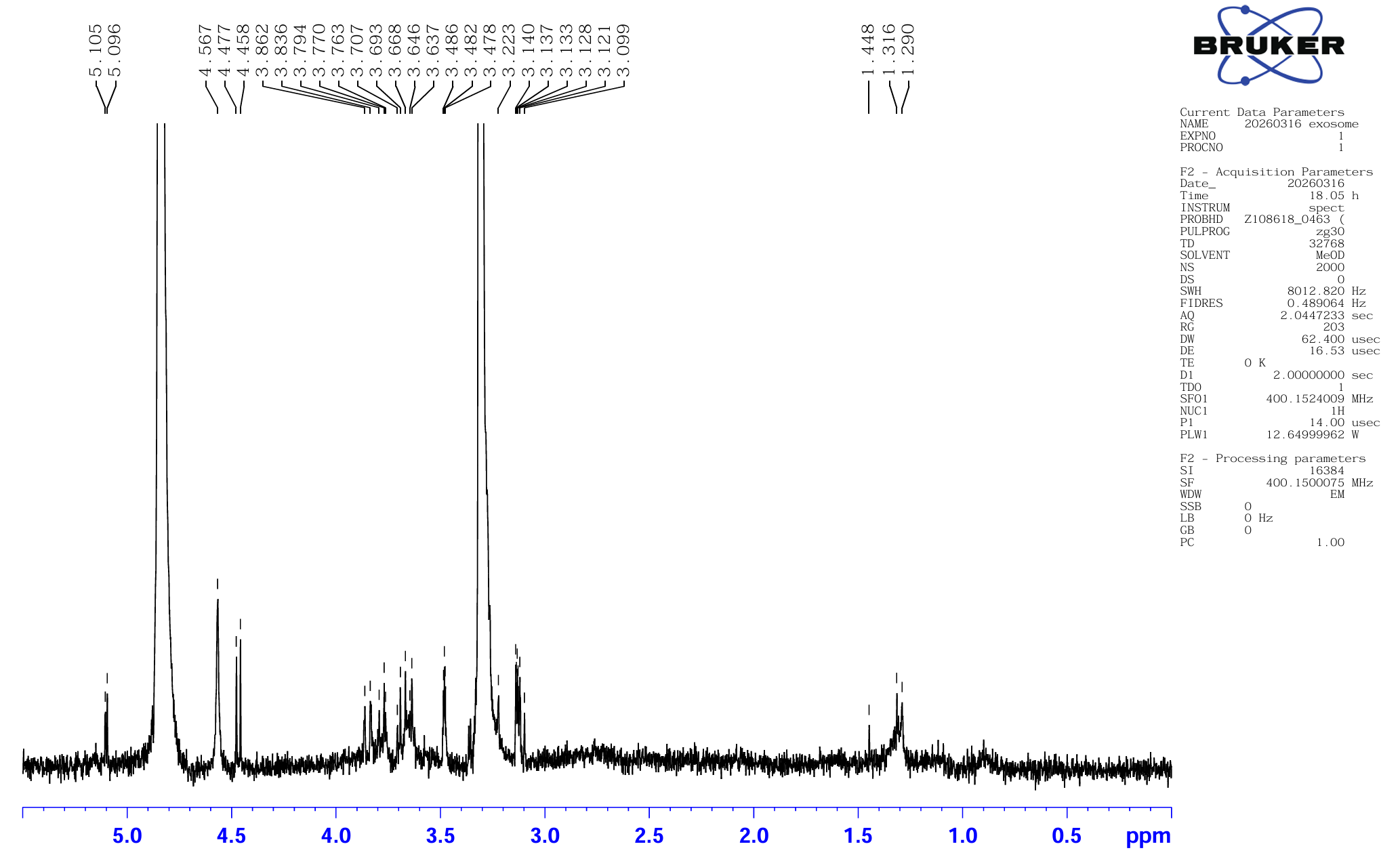


Figure S1. ¹H-NMR spectrum of AZEVs.

The spectrum shows broad carbohydrate-associated resonances (3–5 ppm) and weak, unresolved aliphatic signals, consistent with lipid- and glycan-associated components typically observed in heterogeneous extracellular vesicle preparations.
